# Supplementary material for: Empirical methods for controlling false positives and estimating confidence in ChIP-Seq peaks
Source: BMC Bioinformatics. 2008 Dec 5;9:523. doi: 10.1186/1471-2105-9-523 (PMC2628906; doi:10.1186/1471-2105-9-523)
Supplement: Additional File 1 — A variety of html documents from the USeq web site detailing the available applications, their best usage, output file type descriptions, command line menus, etc. [file 1471-2105-9-523-S1.zip › USeqUserGuides/JavaDoc.zip!/JavaDoc/gata/aligner/package-use.html]

Uses of Package gata.aligner


|  |  |  |
| --- | --- | --- |
| **Overview** \| **Package** \| Class \| **Use** \| **Tree** \| **Deprecated** \| **Index** \| **Help** | |  |
| PREV   NEXT | **FRAMES**    **NO FRAMES**     **All Classes** |  |


---


## **Uses of Package gata.aligner**

| Packages that use gata.aligner | |
| --- | --- |
| **gata.aligner** | For aligning and post processing DNA sequences using BLAST for subsequent import into GATAPlotter. |
| **gata.main** | Main app, utility, and params for GATAPlotter. |
| **gata.plotter** | GATAPlotter primary panel, sliders, tools, and conservation panels. |

Classes in gata.aligner used by gata.aligner

****AlignerInputForm****

****AlignerInputPanel****

****AlignerPreferences****

****AlignParams****

****GATAligner****

****LocalAlignment****

Classes in gata.aligner used by gata.main

****Alignment****

****AlignParams****

****FileFocusListener****

****LocalAlignment****

Classes in gata.aligner used by gata.plotter

****Alignment****

****AlignParams****

---


|  |  |  |
| --- | --- | --- |
| **Overview** \| **Package** \| Class \| **Use** \| **Tree** \| **Deprecated** \| **Index** \| **Help** | |  |
| PREV   NEXT | **FRAMES**    **NO FRAMES**     **All Classes** |  |


---
